# Differentiating “Attachment Difficulties” From Autism Spectrum Disorders and Attention Deficit Hyperactivity Disorder: Qualitative Interviews With Experienced Health Care Professionals

**DOI:** 10.3389/fpsyg.2021.780128

**Published:** 2022-02-07

**Authors:** Barry Coughlan, Marinus H. van IJzendoorn, Matt Woolgar, Emma J. L. Weisblatt, Robbie Duschinsky

**Affiliations:** ^1^Department of Public Health and Primary Care, University of Cambridge, Cambridge, United Kingdom; ^2^Department of Psychology, Education and Child Studies, Erasmus University Rotterdam, Rotterdam, Netherlands; ^3^Department of Clinical, Educational and Health Psychology, Division of Psychology and Language Sciences, Faculty of Brain Sciences, University College London, London, United Kingdom; ^4^Institute of Psychiatry, Psychology & Neuroscience, King’s College London, London, United Kingdom; ^5^Cambridgeshire and Peterborough NHS Foundation Trust, Cambridge, United Kingdom; ^6^Cambridge Laboratory for Research into Autism (CLaRA), Department of Experimental Psychology, University of Cambridge, Cambridge, United Kingdom

**Keywords:** autism spectrum disorder (ASD), ADHD, attachment–a strong affectional bond, assessment, qualitative analysis

## Abstract

**Objectives:**

“Attachment difficulties” is an umbrella term often used to describe various forms of non-secure attachment. Differentiating “attachment difficulties” from autism spectrum disorder (hereafter autism) and attention deficit hyperactivity disorder (ADHD) has been characterized as challenging. Few studies have explored how this happens in practice, from the perspective of professionals.

**Design:**

Qualitative study.

**Methods:**

We conducted in-depth semi-structured interviews with (*n* = 17) healthcare professionals from five NHS Foundation Trusts in the United Kingdom. Participants were recruited using a combination of snowballing, convenience and purposive sampling. Data were analyzed using a thematic approach.

**Results:**

We identified six interrelated themes that might reflect difficulties with differential conceptualization. These include: a clinical lexicon of attachment; approaching attachment with caution; contextual factors; perceived characteristic behaviors; assessing attachment and adjacent supports; spotlighting intervention and dual conceptualization.

**Conclusion:**

Our results indicate some of the ways suspicions around attachment are raised in practice. We advocate for more dialogue between research and practice communities on issues of differential conceptualization. We call for collaboration between a panel of experts consisting of attachment and neurodevelopmental orientated practitioners and researchers, to clarify issues around differentiating between attachment difficulties, ASD, and ADHD.

## Introduction

“Attachment difficulties” including “attachment disorders” has been introduced to characterize various forms of non-secure attachment (National Institute for Health and Care Excellence, 2015). Differentiating child mental health problems related to attachment difficulties from autism spectrum disorders (hereafter autism) or attention deficit hyperactivity disorder (ADHD) has been identified as challenging (Klein et al., 2015; McKenzie and Dallos, 2017). Part of this challenge is that both autism and ADHD are heterogeneous conditions and frequently co-occur with adjacent nosological conditions (e.g., intellectual disability) and transdiagnostic difficulties such as anxiety (Simonoff et al., 2008; Thapar and Cooper, 2016; Lai et al., 2019). Another part of the challenge is that the term “attachment” has relevance to a wide range of behavioral presentations (i.e., from elevated levels of distress when separated from a caregiver to disinhibited behavior toward unfamiliar adults) and experiences of care (i.e., from relatively common insensitive parenting practices to institutional deprivation and maltreatment; Ainsworth et al., 1978; Lyons-Ruth et al., 2009; American Psychiatric Association., 2013).

In the 1950s, John Bowlby used the concept of “attachment” to emphasize the importance of close relationships for the social and emotional development of children, particularly in how they relate to others and develop relationships themselves. He disseminated this perspective to clinicians, other applied professionals and the general public, focusing particular attention on the dangers of parental absence (Duschinsky, 2020). However, from the late 1960s, Bowlby ceased popular writing, and instead focused his energies on the development of a scientific theory of attachment. Here the term “attachment” was used in a technical sense to mean a child’s predisposition to seek access or proximity to key familiar caregivers in response to separation or discomfort (Bowlby, 1969, 1982). In these later writings, Bowlby described the availability of the caregiver as the set goal of the “attachment system”; when this system is activated by conditions of alarm various social (attachment) behaviors (e.g., walking, reaching) can be used to achieve the caregiver’s availability. In infants, normative differences in attachment behaviors are typically characterized using Ainsworth’s Strange Situation Procedure (SSP) (Ainsworth et al., 1978). This assessment takes place in a novel environment containing toys and includes two episodes of separation and reunion with the caregiver. Other methods of assessing attachment include Q-sort methods (Waters and Deane, 1985), story stems in middle childhood (Green et al., 2000), or interviews in later childhood or early adolescence (Shmueli-Goetz et al., 2008). Many coding systems describe four patterns of attachment: insecure-avoidant, secure, insecure-ambivalent/resistant, or disorganized (Ainsworth et al., 1978; Main and Solomon, 1986, 1990).

It is generally accepted that “insecure” and “disorganized” attachment offer insights into the child’s experiences of their caregiver and also represent risk factors for later socioemotional and behavioral difficulties (National Institute for Health and Care Excellence, 2015; Zeanah and Guyon-Harris, 2020). Insecure attachment, particularly the avoidant classification, has been linked to internalizing and externalizing difficulties in later childhood (Groh et al., 2012). Also, disorganized attachment is associated with externalizing behaviors (Fearon et al., 2010), and has been linked to controlling behaviors in later childhood (Main and Cassidy, 1988; Van Ijzendoorn et al., 1999). Indeed, even more broadly speaking, unsupportive maternal behaviors at 8 months have been linked with adult mental health difficulties and medical problems (Fan et al., 2014).

The concepts of “insecure” and “disorganized” attachment stems from research in developmental science, which has been strongly influenced by Bowlby’s later work on the attachment system and reunions. By contrast, the concept of “attachment disorders” stems from psychiatric nosology, influenced by Bowlby’s early work on the potential harms to children of the absence of a familiar caregiver (Duschinsky, 2020).

Current classification systems describe two “attachment disorders”: reactive attachment disorder (RAD) and disinhibited social engagement disorder (DSED) (American Psychiatric Association., 2013; World Health Organization, 2018). Both are anticipated to result from quantitatively substantive episodes of “insufficient care” where opportunities to forge attachment bonds are limited (e.g., institutionalization). This is in contrast to insecure attachment which is anticipated to stem mainly from caregiving based avenues. According to the DSM-5, RAD is characterized by “a consistent pattern of emotionally withdrawn behaviors toward adult caregivers” and “social and emotional disturbances” (American Psychiatric Association., 2013, p. 265). By contrast, DSED behavioral patterns of social disinhibition and lack of differentiation between familiar and unfamiliar adults when seeking help.

In best practice guidance, the developmental and the psychiatric concepts are brought together under the umbrella of “attachment difficulties” (National Institute for Health and Care Excellence, 2015). The extent to which insecure attachment patterns, as opposed to disorganized, are included under the umbrella of “attachment difficulties” is somewhat ambiguous. That is, National Institute for Health and Care Excellent (NICE) guidance indicates that the term “attachment difficulties” refers to disorganized, RAD and DSED. Yet in parts of the full guidance document, it appears that the term has been stretched to include insecure patterns as well, for instance describing insecure rates in discussion of the prevalence. Further complexity is added by debates between attachment communities over the recognition of disorganized attachment.

Applying attachment concepts within the context of autism and ADHD has proved both elucidating and challenging. On the one hand, it has been shown that many children with autism do develop secure attachments to their caregivers (Rutgers et al., 2004; Teague et al., 2017); atypical social communication does not exclude the possibility of experiencing the caregiver as available when needed. Yet there are pockets of symptomatic similarities between the constellations of features which represent all of these conditions. For instance, while stereotypies (e.g., atypical hand movements) are typically coded as an indicator of disorganization when the attachment system is activated (Main and Solomon, 1990), these behaviors are also a well-known marker for autism (American Psychiatric Association., 2013). Meanwhile, the coding of attachment representations holds that a coherent narrative is a crucial marker for secure attachment (Shmueli-Goetz et al., 2008). Yet there is evidence to suggest that this might be difficult for children with ADHD and autism (Lorch et al., 2010; Scholtens et al., 2014; Almeida et al., 2019).

Autism (Rutter et al., 1999, 2007; Hoksbergen et al., 2005; Levin et al., 2015) and ADHD symptoms (Kreppner et al., 2001; Loman et al., 2013) have been found in children who have experienced severe institutional deprivation, which can be anticipated to represent a form of attachment deprivation (i.e., limited opportunities to forge an attachment bond with a consistent caregiver). On the topic of attachment disorders, Sadiq et al. (2012) found that a significant number of children with RAD/DSED scored in the clinical range on the reference standard Autism Diagnostic Interview (Lord et al., 1994). Conversely, Davidson et al. (2015) found that 62% (*n* = 36) of children with autism met core symptom criteria for an attachment disorder according to the Child and Adolescent Psychiatric Assessment for Reactive Attachment Disorder (CAPA-RAD) (Minnis et al., 2009). Experienced clinicians, however, were able to differentiate the presentations after observing the child’s behavior. Regarding ADHD, psychiatric classification systems caution that the socially impulsive behaviors associated with ADHD can resemble the disinhibited behavior, which is characteristic of DSED. Interestingly, data from the Bucharest Early Intervention Study found a significant association between symptoms of ADHD and DSED at, but not before, 54 months (Gleason et al., 2011). Taken together, this literature suggests that there are times when these difficulties can co-occur and also present as symptomatically similar.

The standardized assessment apparatus (e.g., SSP, Q-Sort) used by developmental scientists to assess attachment are often not available to most applied contexts. The reasons for this are likely various, but it is worth noting that many attachment tools require expensive, specialized training. To date, no studies have reported on the experiences of psychological professionals in responding to this predicament. However, qualitative work with social workers indicates there is anxiety about how to draw links between research and assessment (North, 2019).

Recently, there have been several promising attempts to strengthen links between applied and academic communities. These include preliminary work on clinically usable assessment tools (Cadman et al., 2018), interventions (Steele and Steele, 2017), best practice guidance (National Institute for Health and Care Excellence, 2015) and consensus statements (Granqvist et al., 2017). While there is credible evidence for the utility of attachment-based interventions in applied contexts, the relative benefits of the assessment tools compared to assessment-as-usual remains as yet untested. However, efforts are currently underway (e.g., Cooke et al., 2020; van der Asdonk et al., 2020). Still, the absence of consistent assessment tools and the uneven availability of information means that it is unclear whether “attachment” is being used in the same way by clinical and research communities.

The limited availability of applied assessment tools has contributed to difficulties in differential conceptualization. One major attempt to develop a tool designed to codify symptomatic differences between autism and attachment difficulties has been work on the Coventry Grid and the Coventry Grid interview (Moran, 2010; Flackhill et al., 2017). Core to both assessments is the qualitative difference in “emotional feel” between children with attachment difficulties and those with autism. However, the Grid has also been criticized by some attachment researchers (e.g., McKenzie and Dallos, 2017), for problems in the conceptualization attachment difficulties (e.g., combining different attachment patterns).

Qualitative interviews with experienced professionals offer an important window into the application of attachment concepts in assessment, including how factors associated with attachment difficulties are contrasted with the neurodevelopmental differences related to autism and ADHD. This qualitative study aims to provide an account of “attachment” assessment practices and how experienced healthcare professionals differentiate attachment-related issues from neurodevelopmental conditions such as autism and ADHD.

## Materials and Methods

This qualitative study is part of a lager multi-pronged work on neurodevelopmental assessment practices and referral pathways with general practitioners (*n* = 8) and health care professionals (*n* = 17) in Child and Adolescent Mental Health Services (CAMHS). Here we discuss interviews with seventeen healthcare professionals (HCPs) working in five NHS CAMHS services across the United Kingdom regarding their experiences using attachment in a neurodevelopmental context. An information power approach informed our sampling (Malterud et al., 2016). This approach contends that the sample size in qualitative research depends on the following factors: aim, sample specificity, use of established theory, quality of the dialog and analytical strategy. Findings regarding identification, referral pathways, and neurodevelopmental assessment are reported elsewhere. This study focuses exclusively on issues regarding differentiating “attachment difficulties” from autism and ADHD. The set of interrelated aims for the studies described are relatively narrow as they focused on a specific set of conditions (i.e., autism, ADHD, and attachment-related difficulties) and their assessment. The participants belonged to a specific group (i.e., experienced child mental health professionals), and all had at least 3 years of post-qualification experience working with children and families. By recruiting from various services, we were able to explore variation whilst keeping a relatively specific sample. Theoretical background was strong, and the quality of the dialog was enhanced by the interviewers experience working in a neurodevelopmental service. Finally, in terms of analysis, these studies attempt to provide an in-depth analysis of assessment practices.

Participants were recruited using a combination of purposive, snowball, and convenience sampling. We actively sought out different specialist services to obtain a range of perspectives on differential conceptualization. In total, we interviewed HCPs from five NHS Foundation Trusts. Each participant had at least 3 years of post-qualification experience working with children and families. Nine worked in either neurodevelopmental, autism, or core CAMHS; eight worked in specialist CAMHS and neurodevelopmental services. We sought out professionals working in specialist neurodevelopmental service because these services have expertise in diagnosing complex cases. Ethical approval and permissions for the study were obtained from the University of Cambridge Psychology Committee [PRE.2018.019], The Health Research Authority and local NHS research and development teams approved the study before data collection. Written informed consent was received prior to data collection and consent was confirmed verbally at the end of each interview.

To direct the interviews, a topic guide was developed drawing from the available literature and clinical experience of the research team. This guide contained the following sections: professional background; reflections on routine clinical work; hypothetical case study and views on referral pathways. In the larger study, there were two case studies: one for general practitioners, and one for CAMHS HCPS. Here we report only on CAMHS HCPs. Therefore, all participants in this study were asked to read the case study for CAMHS HCPS. Participants were asked to read the case study during the interview. See Supplementary Materials 1, 2 for interview guide and hypothetical case study. Core to the idea of the case study is the idea that participants would draw on their previous judgments when making decisions.

The guide was piloted with three psychologists, and questions were also conferenced with two further academic clinicians. Following feedback from both this piloting and a patient and public involvement group at a local hospital, adjustments were made to the topic guide. All interviews were conducted by [author BC] who is a Ph.D. student and has experience working in a neurodevelopmental service as an assistant psychologist. Interviews took place remotely or face-to-face, and each interview was audio recorded.

Thematic analysis (Braun and Clarke, 2006) was used to identify patterns in the data. Each audio recording was listened to and read twice before coding. Coding was conducted by author one, which we acknowledge as a limitation. Divergent views were actively sought out and considered. This was done by coding the transcripts and searching for counter examples. We regard this as good practice in thematic analysis. Codes were grouped into descriptive and analytical themes and discussed within the research team.

## Results

Our analysis identified the following six interrelated themes: *a clinical lexicon of attachment; approaching attachment with caution; contextual factors; perceived characteristic behaviors; assessing attachment and adjacent supports; spotlighting intervention and dual conceptualization*.

### A Clinical Lexicon of Attachment

As we will discuss, throughout the interviews attachment tended to be used to index contextual factors or ascribe meaning to certain behaviors. One meaning attributed to “attachment” was as a locus of mental health problems. As such, the term was sometimes used interchangeably with terms such as *trauma* or *adverse childhood experiences*. Indeed, attachment was sometimes discussed alongside *developmental trauma*, *PTSD*, and *separation anxiety*.

Beyond the term “attachment” itself, at times, HCPs seemed to draw from the lexicon of academic research by using phrases such as “*secure attachment*,” “*attachment figure*,” “*attachment styles*,” “*insecure attachment*,” “*disorganized attachment*,” and “*ambivalent attachment*.” However, the use of these terms was relatively infrequent across the set of interviews. Cognate concepts such as “safety” and the idea of a safe environment were also identified in some transcripts.

References to unofficial attachment terminology could also be found in some cases, though this was less frequent. This language comprised terms that are not used by attachment researchers, but which nonetheless were used by speakers as if referring to an established body of knowledge. Such expressions included “*solid attachment*,” “*good attachment*,” “*unusual attachment*,” and “*poor attachment*.”

In the example below, one professional describes how they determined that a child with behavioral issues had a “secure” and “solid attachment”:

“*It was quite difficult, but actually the more questions we asked mom very specific questions about how that child operated within the family. I think that pulled out actually some quite secure attachment. So we thought there was attachment issues, but underneath it, we saw where the attachments were secure and actually that was more about issues with parental management and boundaries. But underneath that, there was solid attachment*.” *PTND03*.

*Interviewer: Can you tell me a little bit about those questions that you asked?*

“*We were looking at all the behaviors that were demonstrated, such as grabbing an item and throwing it across a room–taking something and injuring somebody inadvertently. So we looked at whether. what the rationale was for the behavior. So why did that child choose to throw a pencil at somebody? Was it because [they] wanted your attention? Was it an impulsive act that [they] just couldn’t stop? Was it trying to get out of a situation that was too busy and noisy?*” *PTND03*.

In this passage, the phrase “solid attachment” seems to be interchangeable with “secure attachment.” What is unclear from this description is the extent to which the questions elicited information that would be considered “attachment relevant” using the framework from developmental research. One way of interpreting this might be that the child is experiencing some externalizing behaviors in response to behavioral boundaries. Yet there are nonetheless positive aspects of the child’s relationship with their caregivers. This would mean that “solid attachment” is functioning similarly to the concept of “relationship.”

Nonetheless, more common than the use of either research or unofficial attachment terminology was attempts by participants to lean away from technical language when discussing attachment.

### Approaching Attachment With Caution

Participants were typically circumspect when discussing the differences between “attachment difficulties” and “attachment disorders.” Several HCPs explicitly stated that commenting on the distinctions between attachment difficulties and attachment disorders was running up against the limits of their attachment expertise. In general, participants tended to prefer the language of “attachment difficulties” over “attachment disorders.” There was a sense that the label of “attachment disorders” was reserved for children with more severe levels of impairment:

“*I’m quite careful. And I tend to use the word difficulty as a general*… *It’s a bit like learning difficulty/learning disability. Because I think I wouldn’t make a diagnosis of an attachment disorder. I think that goes through the Psychology and Psychiatry teams. And actually, that to me indicates it’s at a higher level really where there’s much more significant and perhaps less reversible. I don’t know maybe there is reversibility. I don’t know*.” *PTND04*.

Two participants, both psychiatrists, expressed a degree of discomfort with the term “difficulties,” as fundamentally too elastic.

“*I also think it’s interesting to know whether somebody had had an attachment disorder diagnosis, which is very specific and refers to a diagnostic criteria other than attachment difficulties often. I don’t know what that [attachment difficulties] means [*…*] I think it’s hard sometimes to understand*… *somebody says an autism spectrum disorder. I know what that means because there’s agreed criteria. My experience is we often get children or young people sent to us with attachment difficulties without there being a combined understanding of what that is. We all have attachment-seeking behaviors*.” *PTND15*.

Also, several HCPs indicated that a degree of caution is required when using “attachment” in case formulations of looked after children. These concerns tended to be about the potential for an attachment conceptualization to silence other difficulties:

“*I think sometimes the name ‘attachment disorder’ can be placed on a young person and maybe then may mask the other more subtle difficulties or presentations the young person is presenting with*” *PTND13*.

### Contextual Factors

Contextual factors occupied a vital role in the conceptualization of a case as presenting with relevant attachment difficulties. There were some suggestions that social trends, such as the prevalence of adverse childhood experiences or trauma in a given population, might make an attachment-related conceptualization more relevant. More frequently, however, family factors tended to raise concerns about attachment. An array of family factors were identified in the discussions of routine clinical work. These were: parental mental health problems (e.g., depression, personality disorder), familial neurodevelopmental conditions (e.g., ADHD), parental drug and alcohol misuse, parental involvement in the criminal justice system, domestic violence, involvement with the care system and parental history of abuse or neglect. Often, HCPs reflected on cases where several of these factors were in-play.

Overall there was a strong sense that at least one discrete adverse experience or pattern of insufficient care was required to warrant clinical suspicion of attachment difficulties:

“*So you wouldn’t expect to see it in a child unless there was some reason, so a mum that’s had a really difficult delivery or she has post-natal depression. That might be something you know OK there’s a link. Or a child that’s been removed [or] put into care. Or there’s been significant domestic abuse or violence or things or neglected parenting. So you sort of have the context behind why you’re seeing it*” *PTND04*.

### Perceived Characteristic Behaviors

Once suspicion of attachment difficulties had been raised, HCPs felt that differential conceptualization was challenging. Phrases such as “*that’s been tricky*,” “*it’s a bit difficult*,” and “*struggle to pick that apart*” were common. The locus of these difficulties was clear in the case of autism and attachment difficulties. Both presentations were felt to share similar social communication atypicalities and some repetitive behaviors (e.g., repetitive conversation around safe topics). By contrast, participants were less clear regarding the characteristic behaviors of attachment difficulties and ADHD.

Much of the time, contextual factors were given particular weight by participants in distinguishing attachment difficulties from autism or ADHD. However, when comparing an attachment presentation to either autism or ADHD, attachment difficulties were held to have some distinguishing behavioral characteristics. First, several participants remarked that atypical or intense emotional responses to separation and particular caregivers were a core feature of a presentation indicating attachment difficulties. In contrast, atypical behaviors that were consistent across contexts were perceived as characteristic of autism or ADHD:

“*It [attachment-related difficulties] can be more confined to one environment than another. Whereas with ADHD and ASD, you expect to see some of it across all environments. I know with ASD it can be more subtle at school and they come out, and they’ll have meltdowns. And it can present a lot like autism. So some difficulties making friendships - making new relationships. [There] can be huge difficulties going out to new places, difficulties going to school because you’re worried about what’s going on at home*.” *PTND10*.

Second, there was a sense that there was a particular intensity to the internalizing or externalizing behaviors associated with attachment difficulties. Participants offered a wide array of examples of these behaviors, including smashing windows, stealing weapons and threatening other children, self-soothing after being reprimanded by a parent, or intense clinging to caregivers. In the example below one participant reflects on what they perceived as a clear expression of attachment difficulties:

“*It was again very clear from the presentation that it’s attachment difficulties*… *from presentation and interaction of and relationship with the mum and child. There was a situation when the child got upset in the session about Mum’s response, and there was a very extreme response from the child, so the child went outside of the clinic room. [The child] sat in the middle of the corridor and tried to soothe themselves*… *without*… *so the child did not go to parent*.” *PTND05*.

Two participants also made explicit references to displays of sequential contradictory behavior as a marker for attachment difficulties:

“*So doing that pushing mum, pulling mum, go away I hate you come back I love you*.” *PTND02*.

Controlling behaviors were identified as characteristic of attachment difficulties by several participants. These behaviors ranged from controlling behaviors toward caregivers to controlling other children in school:

“*And [the child] took on the role [they] wanted to play Mummies and Daddies. So [they] said who everybody was and the way that [they] portrayed themselves as the mum was very disciplinarian and controlling [.]. And said, ‘You must not speak.’ And [they] started getting almost quite aggressive with the young person [they were] playing with. Saying, ‘No I told you not to speak,’ and really enacting quite a punitive parent and then afterward took on a teacher role and similarly very much took charge*.” *PTND08*.

However, controlling behaviors were not exclusively associated with attachment difficulties:

“*[the child] was holding her baby sibling*… *[*…*]*…*that kind of warmth and responding to the baby wasn’t there it was much more about directing behavior and what you must and must not do so yeah that came across as much more an attachment difficulty in the play than we would typically see. You know children with social communication difficulties do sometimes want to take charge of a situation*…*[*…*]. but it just seemed to tip over into something much more anxious and came across as being quite aggressive. As though she was really getting quite distressed when she was taking on these roles*.” *PTND08*.

Other behaviors variously linked by participants to attachment difficulties were: disinhibited behavior, hypervigilance, interpersonal difficulties, problems with behavioral boundaries, eating problems, imaginary friends, perfectionism, callous-unemotional behavior and toileting issues. Indeed, overall there was extensive variation regarding the behaviors considered attachment-related.

In response to the case study, several participants identified displays of approach-avoidance conflict as a possible marker for attachment difficulties. However, other participants acknowledged that this could be unrelated to attachment. They also identified hand-flapping as a marker for autism rather than attachment difficulties:

“*hand flapping, which you don’t really see outside of autism*.” *PTND09*.

### Assessing Attachment and Adjacent Supports

Clinical interviews, standardized tools (e.g., ADOS; ADIR; Connor’s Rating Scales; QB Test), and observations of the child across different settings (e.g., home and school) were a routine part of the neurodevelopmental assessment. Clinical interviews about family histories and genograms revealed background information that was perceived as relevant to attachment. Meanwhile, behavioral expressions of attachment difficulties tended to be identified through structured and unstructured neurodevelopmental observations and play-based assessments. Observations at school and home were also an opportunity to see how the child responded to separations and the consistency of behaviors across contexts. Mostly, attachment was assessed through non-standardized methods:

“*So I don’t generally*… *Well, I don’t use formal attachment to assessment tools myself. Not for any amazing reason. So I wouldn’t say it’s not my business or not my training because I think that’s easy to say. But I don’t sort of sit down and assess it in a very formal operationalized way. But I’ll think about all the different dimensions of how secure he is what he does and leave the room. What he says on his own. Reports from school and home about whether he seems to be rejecting or anxious*” *PTND17*.

Several participants highlighted that their services were not commissioned to assess attachment formally. Nevertheless, seven participants did mention the Coventry Grid as part of the differential conceptualization. The extent to which this tool was routinely used was unclear. Some participants, for instance, had difficulty recalling the name of the instrument. Overall, there was a sense that the tool could aide differential conceptualization yet should be used with a degree of caution:

“*I’ve only used it [Coventry Grid] a couple of times. I think there’s not a huge amount of research on it. There are a couple of papers on it, and it’s very clear you should be doing it alongside an ADI [.]. Not just ‘oh well I’ll pull this out the drawer, and I’ll do this, oh gosh you haven’t got autism.’ I think it’s for those ones where like this it could be either or both. That sometimes it’s quite helpful just to help you separate which of these features are the autism which might be attachment*.” *PTND02*.

Often participants drew on multi-agency and cross-disciplinary support once suspicions of attachment difficulties were aroused. Social work, safeguarding teams and specialist attachment services were among some additional supports. This support was sought through formal (e.g., referral) and informal (e.g., case discussions) channels. There was some variation regarding the perceived availability and accessibility of specialism from specialist attachment services. Here one participant in a specialist neurodevelopmental service reflects on her experience working with colleagues in the attachment teams:

“*So we all know each other, and we can do case presentations to that group. And discuss cases, but if we have a case we’re concerned about, or they have one they’re concerned about it’s really just a question of finding each other and just saying would you mind looking at this ADOS for me? Or would you mind talking through this case we don’t always necessarily formally refer over, but we do consult to each other*.” *PTND09*.

In some contrast, another participant described the in a local mental health service described the availability of specialist attachment services “*a disaster, nationally*” *PTND05*.

### Spotlighting Intervention and Dual Conceptualization

Participants indicated that it might be beneficial to start intervention and table or delay diagnostic decisions. References to interventions included *evidence-based parenting groups*, *systemic work*, and *dynamic psychotherapy*. It was reasoned that changes in the child’s environment and support for parents were the priority. However, HCPs also felt that attachment difficulties could be distinguished in part based on whether the child’s symptoms abated following improvement in the child’s caregiving environment. For example:

“*Probably for such a young case I’m not sure I will give a diagnosis of anything. I will probably try and see when we start to work therapeutically with a child and see how it works.[*…*]*…*I will probably try to combine something which is a bit more of a dynamic psychotherapy for the child, and the parents work alongside it*.” *PTND06*.

“*If the environment becomes more containing, and then the child still has clear symptoms of ADHD, I would be inclined to treat it, but not everybody agrees with me. But only after we were really confident that the environment was more containing them a proper boundaries seem to be better at home and at school*” *PTND17*.

However, one participant suggested that the intervention work which empathizes relationship-building might not always be unhelpful for some children with autism.

A common refrain was that children could have attachment difficulties together with autism or ADHD. Under such circumstances, it might not be possible to give a firm diagnosis one way or another, since varied etiological factors might contribute to non-specific symptoms like emotional dysregulation. One consideration around dual conceptualization, however, was the power of a neurodevelopmental diagnosis to divert attention away from relational factors:

“*What we were worried would happen was that they would chop off the trauma bit and leave out that kind of attachment side of it and just have autism. And that mightn’t always be the most useful thing, so the wording of that was very important*” *PTND07*.

## Discussion

As has been observed elsewhere (Sadiq et al., 2012; Klein et al., 2015; McKenzie and Dallos, 2017), assessing autism and ADHD within the context of possible attachment difficulties can be challenging. As found in previous work (e.g., Keddell, 2017), we identified some use of attachment terminology in ways that depart from the discourse of the research community (e.g., “good attachment”). In these instances, attachment language seemed to be invoked, as in Bowlby’s early work, to generally signal the importance of children’s close relationships for their mental health and wellbeing. We also identified some use of the language of attachment research (e.g., “disorganized attachment”). Nonetheless, explicit references to concepts such as “secure” or “insecure attachment” were relatively sparse, and we identified no references to the “attachment system” whatsoever. As Duschinsky (2020) has also observed in a study of literature about attachment directed toward clinicians, in our data the technical theory of attachment research appears not to have traveled as easily or as well as Bowlby’s earlier and simpler account and use of language. Indeed, Bowlby tended to adjust his discourse on attachment, foregrounding certain claims while backgrounding some caveats, depending on his audience.

Even more striking in our data than how attachment concepts were used, was the hesitancy with which they were used. This might be partly explained by the fact that practitioners are accustomed to synthesizing technical information and presenting it in an accessible manner and thus might avoid technical terms from developmental science. Nevertheless, in agreement with previous qualitative research with social workers (North, 2019), HCPs in our sample took a cautious approach toward attachment. We would speculate that more collaborations and dialog between academic and clinical communities would help smooth out some of the divergences between these discourses. This, we anticipate, would fortify correspondence between clinical claims regarding attachment and empirical findings from social, clinical and developmental psychology.

Despite caution among participants, our data does offer some indications into how experienced practitioners sought to differentiate attachment difficulties from autism and ADHD. Most often, clinical suspicions for attachment difficulties were raised by identifiable factors suggesting the lack of a stable caregiver, or a very adverse caregiving environment (e.g., due to serious parental mental health problems). Indeed, many if not all of these factors have been, to different degrees, associated with increased rates of non-secure attachment (Fearon and Belsky, 2016; van IJzendoorn and Bakermans-Kranenburg, 2019). Yet research from developmental psychology also overwhelmingly indicates that a complex network of social, psychological, and genetic factors shapes attachment bonds. Further, there are significant gaps in the current understanding of the intergenerational transmission of attachment (Scheper et al., 2019). The practice challenge was identifying to what degree did contextual factors influence the child’s presenting behaviors. On this topic and echoing sentiments expressed elsewhere (e.g., Woolgar and Baldock, 2015), some participants expressed concerns about how some of these contextual factors might divert attention away from neurodevelopmental explanations. Thus, the salient practice challenge seems one of determining how to weigh these contextual factors; an area on which developmental literature can offer only limited guidance.

One intriguing finding was that relatively everyday caregiving practices (e.g., difficulties in establishing behavioral boundaries) were not prominent in the data. This is in contrast to developmental attachment research, which has indicated that there are numerous pathways to non-secure attachment (Ainsworth et al., 1978; Fearon and Belsky, 2016). A reason for this might be that participants tended to reflect on more complex cases where they perceived the attachment difficulties to be more extensive. Another possible explanation is that, although everyday parenting challenges are linked to non-secure attachment in children, there was a sense that these practices or experiences did not in and of themselves lead to symptoms that resembled symptoms of autism or ADHD.

Although differential conceptualization was, at times, perceived as difficult, participants underlined several important qualities of attachment difficulties. Some of these proposals were not in line with the conclusions of researchers. For instance, difficulties around food do not align with developmental or psychiatric descriptions of attachment and may reflect a conflation between maltreatment and attachment difficulties (Prior and Glaser, 2006).

By contrast, other proposals for distinguishing behaviors were more aligned with the research literature on attachment. For example, some participants indicated that intense or atypical responses to separation from the caregiver signified potential attachment difficulties, whereas, there was a sense that atypical behaviors consistent across contexts seemed to be more readily linked to autism and ADHD. This finding is consequential as it juxtaposes two of the core assumptions undergirding attachment difficulties (i.e., bonds are relationship-specific and reunions after separation are a window into attachment behaviors; Cassidy and Shaver, 2016) and a core prediction about neurodevelopment differences (i.e., some consistency of behaviors across contexts; American Psychiatric Association., 2013; World Health Organization, 2018). This heuristic, however, is perhaps less robust for attachment disorders which are perceived as more pervasive in terms of relational context.

Additionally, there was also a sense that the internalizing and externalizing difficulties associated with attachment difficulties had a different intensity than those typically associated with autism or ADHD. The association between some attachment difficulties and later internalizing and externalizing problems, including controlling behaviors toward caregivers has been documented (Van Ijzendoorn et al., 1999; Fearon et al., 2010; Groh et al., 2017). Yet the extent to which the quality of these behaviors are in and of themselves differential when considering autism or ADHD is a matter requiring further investigation.

Another interesting finding was that hand-flapping was treated by participants as a particularly strong marker for autism. It is true that stereotypies such as hand-flapping are treated as an index of disorganized attachment (Main and Solomon, 1986, 1990); yet when classifying attachment disorganization in children with suspected neurodevelopmental difficulties, it is a coding convention to either forego coding the indices of stereotypies (Willemsen-Swinkels et al., 2000) or to classify these as attachment behaviors only when they appear at critical moments in the SSP, such as reunion, when the coder can be sure the attachment system is activated (Rozga et al., 2018). Thus, it seems that across developmental and clinical communities, there is an acknowledgment that hand flapping is more characteristic of autism than attachment, especially if it occurs regularly outside of contexts in which a child wishes for comfort. Flapping can occur as an emotional overflow movement, and is not viewed as strongly indicative of autism spectrum disorder (ASD) on its own, while other stereotypies are more specific to ASD (flicking fingers while watching out of the corner of the eye, for example).

The data indicate that attachment difficulties tended to be assessed through non-standardized means including naturalistic observations of the child (e.g., in a nursery or the clinic) and interviews with caregivers. This makes sense given that standardized attachment measures and training in them are rarely available to clinicians. Several practitioners indicated that their services were not commissioned to conduct formal attachment assessments. Thus, when deciding to what degree the child’s difficulties were attachment-related or neurodevelopmental participants were reliant on non-standardized approaches (e.g., observing separations at nurseries) and, where available, support from adjacent teams.

Observations of naturalistic reunions and separations are recommended in some of the guidance material as an important window into attachment bonds (Zeanah et al., 2011). Yet the data both limited and mixed. For example, some early work shows little or no association between the SSP conducted at home versus in a traditional laboratory setting (Goossens et al., 1986). Recently, however Bick et al. (2012) has found evidence to suggest that child behaviors during natural reunions at nursery correlate with secure attachment classifications in the SSP. Some preliminary work indicates that shorter and thus more clinically feasible adaptations of classic research measures assessment tools such as the Brief Attachment Scale (Cadman et al., 2018) show promising psychometric properties when compared to established attachment measures. Importantly, however, many attachment tools have not yet established sufficient specificity and sensitivity to be used in clinical work for individual level conceptualization. Indeed, the standardized assessment of attachment difficulties in clinical practice is topic on which psychometric work has, to a large extent, fallen wide. If attachment disorders were more common, or insecure attachment a recognized medical diagnosis, there might have been more impetus and funding for the development of such tools. However, over the decades, there has been little institutional support for research-practice links in the assessment of attachment for the purpose of individual diagnosis.

For several participants, the Coventry Grid (Moran, 2010; Flackhill et al., 2017) seemed to provide some guidance when differentiating autism from attachment difficulties. Indeed, some participants echoed a core premise of the Grid in that attachment signaled difficulties with emotional regulation. A recent conceptual analysis and review of attachment, autism and ADHD, (Coughlan et al., in review) compared items from the Coventry Grid with those in the Attachment Q-Set (Waters, 1987) and the Disturbances of Attachment Interview (Smyke and Zeanah, 1999). This comparison concluded that while the Coventry Grid might offer some help in distinguishing autistic and non-autistic behaviors, several of the items treated as characteristic of attachment difficulties were discrepant with attachment theory as it is rendered in the developmental literature.

We envisage that improving links between research and clinical practice would benefit both communities. Some of the behaviors the experienced practitioners in our sample regarded as characteristic of attachment difficulties are discrepant with the theory and findings of researchers; others have not been empirically examined. Although the availability of standardized assessments might be limited, applied practitioners have access to various forms of implicit and explicit information about the children and families in their care. This was evident in many of the reflections on routine clinical work in the current study. We anticipate that this practical knowledge will prove an essential asset in any future efforts to validate assessments for attachment difficulties that can be delivered in applied contexts with sufficient sensitivity and specificity.

Furthermore, several participants spotlighted the importance of examining a child’s response to supportive intervention with their caregivers as a means of appraising the extent to which their symptoms stem from attachment difficulties. Like medication might sometimes be prescribed by clinicians in part as intervention, but in part as a strategy for diagnostic differentiation (“pharmacological dissection”), it may be that the attachment research community could resolve theoretical questions by seeing how children with likely attachment difficulties respond to different forms of clinical intervention. Recently, van der Asdonk et al. (2020) have pioneered work along these lines; we are encouraged to see that the use of intervention as a means of assessing for attachment difficulties finds support in the practice of experienced clinicians.

Distinguishing attachment difficulties from neurodevelopmental differences is a microcosm of the broader clinical challenge of differential conceptualization and comorbidity. In our view, there are two lines of empirical inquiry on this topic which would merit priority. First, as described above, some participants indicated that there could be varied etiological factors contributing to symptoms like emotional dysregulation. This conclusion is in line with a proposal by Caspi et al. (2014) who have argued that diverse adversities can contribute to the likelihood of mental ill-health in general. Rather than focusing on diagnostic categories, they proposed that mental ill-health might better be conceptualized in terms of a general psychopathology (hereafter *p*) factor, i.e., a grand latent factor underpinning the symptoms usually differentiated into conditions such as autism, ADHD, and attachment difficulties (Neumann et al., 2016). A general *p* factor would account for the high rates of comorbidity between neurodevelopmental conditions (Saito et al., 2020), the absence of biomedical markers, and might partly explain why children with autism and ADHD have higher rates of non-secure attachment compared to neurotypical peers (Rutgers et al., 2004; Storebø et al., 2016; Teague et al., 2017). It would be valuable to examine the extent to which clinical practice explicitly organized in terms of the *p* factor rather than diagnoses would be feasible.

Second, our participants repeatedly emphasized that the conditions under discussion are notably heterogeneous. Many of our participants emphasized that they tend to focus attention on symptoms rather than diagnostic entities. In line with this, a network approach (Borsboom and Cramer, 2013) might yield valuable insights in terms of identifying the clusters of symptoms that are shared amongst these conditions as well as pointing to symptoms that are not shared between these respective constellations. For instance, it might be the case that specific networks of attachment symptoms resemble some but not all networks of autism or ADHD symptoms. This would mean that the oft-cited overlap between these conditions applies to a particular manifestation of the conditions rather than the entire constellation. The insights of clinicians will be essential to the meaningful interpretation of such clusters of symptoms, and advancement from the identification to the design of interventions. Recently we have used the network approach to explore socioemotional profiles in children and young with autism and ADHD (Coughlan et al., 2021). However, due to the sample size we were unable to compare networks for children with attachment disorders. Future work, therefore, might explore the broader category of “attachment difficulties” and compare these symptoms networks with a group of children with autism and ADHD, respectively.

Finally, as others have noted (Davidson et al., 2015) there is a keen clinical interest in the differential conceptualization of attachment and neurodevelopmental conditions. Therefore, we suggest that bringing together an interprofessional panel of experts on these issues would be a positive next step in terms of progressing this conversation. We envisaged that it would be important that this panel is comprised of frontline healthcare professionals, developmental scientists, clinical researchers, service managers, and experts by experience. This would help clarify definitions as well as identifying symptomatic similarities and considerations around different assessment tools, and might lead to a coordinated research agenda for this complex area of inquiry and clinical work.

### Strengths and Limitations

A strength of this study is that it included professionals across the assessment pathway, many of whom would be considered experts in neurodevelopment. Also, interviews were in-depth and explored a range of issues relating to assessing these conditions in children. Indeed, it would have been valuable to contrast some of the views of the professionals in this study with those working in specialist attachment services and attachment researchers. This is a limitation we hope is addressed in future research. Another possible limitation is that the research group conducting the study may have been known to participants for our interest in attachment. Some of the participants may have adjusted their responses based on perceptions about our research group. For instance, participants might have been more hesitant to use certain attachment terminology or comment on the differences between attachment disorders and attachment difficulties given the context. However, the participants, as evidenced by training and experience, occupied a status higher than the first author and interviewer, a doctoral student. Finally, transcripts were coded by author one who brought preliminary themes for discussion with the research group. Therefore, we did not have second coding on the transcripts which we acknowledge as a limitation. Emergent findings from all aspects of qualitative study were sent to participants however we did not conduct extensive member checking. This we also regard as a limitation.

## Conclusion

This study spotlights some of the challenges and methods of distinguishing attachment difficulties from autism and ADHD in practice. There were times where the phrases used to discuss attachment did not track with developmental accounts, and thus it was unclear whether “attachment” was doing the same work across contexts. Contextual factors increased suspicions that attachment difficulties might be at stake, including parental mental health problems, domestic violence, abuse and neglect. There was a sense that each of these conditions could co-occur. We call for a panel of experts consisting of researchers and practitioners to clarify issues around definitions, symptomatic overlaps and divergences, assessment tools, and a coordinated research agenda.

## Data Availability Statement

The datasets presented in this article are not readily available because although all participants were reminded not to disclose any personally identifiable information about patients or families, the transcripts do include reflections on routine clinical work and service arrangements. Thus, to further safeguard the privacy or the participants and those involved in their services, we cannot make the transcripts available. Requests to access the datasets should be directed to BC, (bc471@medschl.cam.ac.uk) for further information about the data.

## Ethics Statement

The studies involving human participants were reviewed and approved by the Psychology Ethics Committee at the University of Cambridge [PRE.2018.019]. The patients/participants provided their written informed consent to participate in this study.

## Author Contributions

BC, MW, MHvIJ, and RD conceptualized the study and contributed to the analysis. EW and BC contributed to recruitment. BC wrote the first draft of the manuscript. All authors reviewed the manuscript.

## Author Disclaimer

The views expressed are those of the authors and not necessarily those of the NHS, the NIHR, or the Department of Health.

## Conflict of Interest

The authors declare that the research was conducted in the absence of any commercial or financial relationships that could be construed as a potential conflict of interest.

## Publisher’s Note

All claims expressed in this article are solely those of the authors and do not necessarily represent those of their affiliated organizations, or those of the publisher, the editors and the reviewers. Any product that may be evaluated in this article, or claim that may be made by its manufacturer, is not guaranteed or endorsed by the publisher.
