# Supplementary material for: Differentiating “Attachment Difficulties” From Autism Spectrum Disorders and Attention Deficit Hyperactivity Disorder: Qualitative Interviews With Experienced Health Care Professionals
Source: Front Psychol. 2022 Feb 7;12:780128. doi: 10.3389/fpsyg.2021.780128 (PMC8860234; doi:10.3389/fpsyg.2021.780128)
Supplement: Supplementary file 2 [file Data_Sheet_2.docx]

#### Supplement 2: Case Study Hypothetical Case Study (For CAMHS)

Robert (aged 6) was referred to CAMHS by his general practitioner due to concerns regarding his behaviour and overall development. Robert has made little progress, academically, since starting school one year ago and is described as having few friends. In addition, he has been growing increasingly aggressive towards his mother, Linda (33), and recently threw her laptop computer at a wall. According to Linda, his behavioural issues are longstanding, and she is finding it difficult to manage. Previously there have been safeguarding concerns and social work has been involved with the family.

A developmental history questionnaire revealed no significant issues with pregnancy or birth. Under the strengths section of the questionnaire, “none” was written. Robert achieved his motor milestones; however, his language development was delayed. Previously he received speech and language therapy in the community. The questionnaire also revealed that Robert has an older half sibling, Chris (14) who has a diagnosis of ADHD. The referral was accepted on the basis of the paper screen.

In the first instance, Robert was booked in for an unstructured behavioural/play assessment and offered a place on a parenting programme. Prior to the assessment, the team received a number of phone calls from Robert’s father, Tim (32). In the first phone call Tim said he would not attend the parenting course as parenting isn’t the issue and that he found the invitation insulting.

Robert was on the waiting list for just over 3 months. Over this period, Tim phoned the team a number of times to express his dissatisfaction with the waiting list. At one-point Linda phoned crying saying Robert was “out of control”.

One month later Robert attended the assessment. From the outset, Robert appeared distressed (i.e. crying). Linda made numerous attempts to comfort Robert, but he moved away in response to each of her approaches. At one-point Robert kicked out at Linda. There are what look like two distinctive episodes of hand-flapping.

During the assessment Robert presents as active (e.g. jumping up and down, throwing ball in the air) and moved from one activity to another in quick succession. Robert’s eye contact was fleeting, and he did not demonstrate the full range of facial expressions. In terms of conversation, Robert spoke in complex sentences, although the subject matter was a little repetitive and mainly around his favourite toy (Shopkins).

After the assessment, Tim and Linda completed the autism diagnostic interview (ADIR). The assessment lasted 2hrs 30 mins. When asked about development prior to three years, Linda disclosed that Robert lived with his grandmother beginning when he was 18 months old to just after his third birthday, as Linda and Tim were separated during this period. During the separation, Linda was an inpatient at a local mental health facility. Robert’s score on the ADI-R was just below the threshold for autism.
